# Pathogenesis of uterine diseases in dairy cattle and implications for fertility

**DOI:** 10.21451/1984-3143-AR2018-0023

**Published:** 2018-08-03

**Authors:** Marc Drillich, Karen Wagener

**Affiliations:** University Clinic for Ruminants, Clinical Unit for Herd Health Management in Ruminants, Department for Farm Animals and Veterinary Public Health, University of Veterinary Medicine (Vetmeduni), Vienna, Austria

**Keywords:** endometritis, metritis, microbiota, uterine defense mechanism, uterine disease.

## Abstract

Uterine diseases in cattle occur at all stages of the reproduction cycle but the majority of cases is found in the postpartum period. The inflammation of the uterus is generally defined as metritis or endometritis, with several graduations, e.g. puerperal metritis, clinical metritis, clinical or subclinical endometritis. Whether uterine diseases have a negligible, moderate or detrimental effect on fertility is still under discussion and depends on definitions and classification. In the past, it was assumed that the pregnant uterus is free of pathogens, but recent studies found several species including pathogens in the uterus and endometrium of pregnant cows. After parturition, a broad diversity of bacteria with >200 different species has been found in the early postpartum period. Not all of these bacteria, however, are considered as pathogens. Furthermore, bacteriological findings provide only evidence for infection but not for inflammation. For some bacteria, particularly *Escherichia coli* and *Trueperella pyogenes* pathogenic mechanism resulting in metritis and endometritis have been elucidated in detail. The role of bacteria that can be regarded as opportunistic or potential pathogens, e.g. *Bacillus pumilus*, is still under investigation. The understanding of the uterine microbiota and its interactions is increasing with the use of modern high-resolution techniques such as Fourier- transform infrared spectroscopy. Endometrial cytology provides additional information about alterations in the endometrium. Knowledge of innate uterine defense mechanism in cattle has increased a lot in the recent past. It can be speculated that improving or modulating uterine defense mechanism will be part of future prevention and treatment approaches beyond the use of antimicrobials. In this context, cellular and molecular defense mechanisms have been in the focus of interest, e.g. the role of interleukins or mucins. This review gives a short overview on some aspects of recent research on uterine diseases in cattle.

## Introduction

Reproductive performance of cows and heifers is one of the key parameters reflecting the economic success of dairy farming. Several fertility parameters can be used to describe negative effects of uterine diseases on reproductive performance, e.g. days to first service, estrus detection rate, and probability of pregnancy per insemination. Days to first service and estrus detection rate are mainly affected by management (time for estrus detection, accuracy of estrus detection, claw trimming, and several other aspects related to management) and to lesser extent by cows’ diseases, e.g. cysts, conception risk is strongly related to uterine health. Thus, the prevention of uterine diseases, sufficient detection of affected cows and efficacious treatment strategies are important aspects of the fertility management of dairy cows.

Inflammation of the uterus, defined as metritis or endometritis is one of the most common disorders in the postpartum period of dairy cattle. Puerperal metritis is characterized by abnormal discharge, enlarged uterus, dullness, and a rectal temperature >39.5˚C (Sheldon *et al*., 2006). Puerperal metritis is also referred to as acute metritis, toxic puerperal metritis or septic metritis and occurs usually within the first 10 days after parturition.

Clinical or chronic endometritis is defined by the occurrence of purulent or mucopurulent vulvar discharge detected more than 3 weeks postpartum. In contrast to acute metritis, chronic endometritis is not associated with elevated temperature and the animals do not show general signs of illness or a depressed attitude. It has been suggested to perform a clinical examination for endometritis later than 21 or 26 days postpartum. An earlier examination results in a greater proportion of false-positive diagnoses (LeBlanc *et al*., 2002). Although the term endometritis is well established for cows with vaginal discharge, there is clear evidence that the origin of pus in the vagina is not always the endometrium but could also be the cervix or vagina. Furthermore, Dubuc *et al*. (2010) found a poor agreement between cytological endometritis diagnosed by uterine cytology and vaginal discharge. Thus, Dubuc *et al*. (2010) suggested the term purulent vaginal discharge (PVD) as a more accurate description than clinical endometritis. The authors of this paper, however, prefer to follow the classical rules of medical terminology for a disease and not to use symptoms as synonymous for a diagnosis.

For the definition and diagnosis of subclinical or cytological endometritis, cytological samples are taken from the endometrium to determine the percentage of polymorphonuclear cells (PMN) in the smears (Kasimanickam *et al*., 2004). A proportion of 5% PMN can be regarded as diagnostic for subclinical endometritis (Madoz *et al*., 2013). Research on subclinical endometritis and diagnostic techniques has been recently reviewed by De Boer *et al*. (2014) and Wagener *et al*. (2017a).

## Uterine infection, uterine microbiota

The contamination of the uterus after parturition with a broad diversity of bacteria is regarded as inevitable. This process with contamination, elimination and subsequent re-contamination is complex and not fully understood (Földi *et al*., 2006; Wagener *et al*., 2015). This concept becomes even more complex with the fact that the pregnant uterus is not sterile, as thought for many years, but may be colonized with several bacterial species including pathogens such as *Trueperella pyogenes* (Karstrup *et al*. 2017; Moore *et al*. 2017). These findings open a new discussion about long-lasting effects of uterine infections but also on pathogenic mechanism of bacteria, i.e. under which conditions an infection has detrimental effects - or not - on conception and pregnancy. Furthermore, there is some evidence that bacteria may also invade the uterus via blood, e.g from the gut (Jeon *et al*., 2017). Whether natural defense mechanisms eliminate the majority of invaded bacteria or clinical diseases manifest depends on the bacterial load and pathogenicity of bacteria as well as on the immune status of the cow (Sheldon *et al*., 2002; LeBlanc *et al*., 2011; Jeon *et al*., 2016). Thus, the occurrence of metritis and endometritis is not only a question of the invading bacterial species. Understanding these interactions and the changes in bacterial composition of the uterine microbiota prior to the appearance of clinical signs of a disease may play a crucial role for the development of optimal prevention and intervention strategies in the future (Madoz *et al*., 2014).

Persistent bacterial infection of the uterus may cause metritis and endometritis. *Escherichia coli, T. pyogenes, Fusobacterium necrophorum* and *Prevotalla spp*. are common intrauterine pathogens (Földi *et al*., 2006; Sheldon *et al*., 2006), although the bacterial virulence factors involved in uterine pathology and the exact mechanism of bacterial pathogenicity are largely unknown (Bicalho *et al*., 2012). In the recent past, however, the effects of uterine infections with *E. co*li and *E. coli*-derived endotoxins lipopolysaccharides (LPS) have been studied and described in detail (Williams *et al*., 2008; Sheldon *et al*., 2009, 2010; Herath *et al*., 2009; Goldstone *et al*., 2014). It has been shown that LPS, a component of the bacterial membrane, has detrimental effects on the endometrium, disrupts uterine and also ovarian function, but also plays role in the innate immune response. One mechanism is that Toll-like receptors (TLR) on the endometrial cells bind LPS, leading to secretion of cytokines and chemokines. Chemokines attract neutrophils and macrophages to eliminate bacteria. There is evidence that a specific strain of *E. coli*, EnPEC, is the main pathogen for metritis (Sheldon *et al*., 2009).

Whereas *E. coli* can be regarded as one of the major pathogens associated with early postpartum metritis, *T. pyogenes* is supposed to be responsible for endometritis later in the puerperium. In the past, preceding infections with *E. coli* were assumed to facilitate subsequent persistence of *T. pyogenes* infections (Dohmen *et al*., 1995, 2000). Recent studies, however, did not support this hypothesis (Prunner *et al*., 2014; Wagener *et al*., 2014b). In contrast, a positive correlation between occurrence of *Streptococcus uberis* on day 3 postpartum and subsequent *T. pyogenes* infections was found, which is in line with previous studies reporting an increased risk of purulent vaginal discharge related to the presence of α-hemolytic Streptococci (Werner *et al*., 2012). Furthermore, it was shown that specific subtypes of *S. uberis* were associated with the uterine health status of postpartum dairy cows (Wagener *et al*., 2014b).

A study from Amos *et al*. (2014) provided evidence that the exotoxin pyolysin (PLO) is the major virulence factor of *T. pyogenes*. Interestingly, endometrial stroma cells were more sensitive to PLO mediated cytolysis than epithelial cells. Thus, it seems that detrimental effects of *T. pyogenes* at the endometrium occur once the epithelium layer is disrupted after parturition. However, the PLO gene is present in all *T. pyogenes* strains and recombinant PLO alone did not stimulate a host inflammation response. Thus, further research should elucidate if the cellular response, that is commonly associated with bacterial infections, is triggered by other virulence factors of *T. pyogenes* (Bicalho *et al*., 2012) or by co-occurring other intrauterine bacterial species.

Beside *E.coli* and *T. pyogenes*, a broad variety of other bacterial species can be found in the bovine postpartum uterus (Földi *et al*., 2006; Prunner *at al*., 2014; Wagener *et al*., 2015). Particularly, the application of high-resolution techniques brought deep insights into the uterine microbiota. Metagenomic studies (Santos *et al*., 2011; Santos and Bicalho, 2012) and analyses performed with the aid of chemometric- assisted Fourier-transform infrared (FTIR) spectroscopy (Prunner *et al*., 2014; Wagener *et al*., 2015) indicated that the bovine uterine microbial community is diverse, highly dynamic and even much more complex than previously thought. In one of our studies, we could show that the aerobic uterine microflora comprised a huge diversity of bacteria belonging to 202 different species, representing 76 genera (Wagener *et al*., 2015). Members of the genus Bacillus, Streptococcus, Enterococcus and coagulase negative staphylococci (CNS) were the most frequently isolated intrauterine bacteria, and have been discussed as potential pathogens or opportunistic contaminants earlier (Westermann *et al*., 2010; Werner *et al*., 2012). On species level, the uterine microflora was dominated by *T. pyogenes, E. coli, Staphylococcus xylosus, B. pumilus* and *S. uberis*. It is interesting to see that the microbiota did not change only over time but showed also differences between cows with different uterine health status (Wagener *et al*., 2015). Additionally, known pathogens, e.g. *T. pyogenes* were identified not only in cows with endometritis but also in clinically healthy cows. Further attention should focus on identifying and characterizing opportunistic pathogens that may synergistically interact with *T. pyogenes.* Detailed information on potential interactions and the interpretation of these findings needs some more investigation.

To gain a deeper insight into mechanism of intrauterine infections it is essential to elucidate the role of bacteria that may act as a mediator of inflammatory host response. Recent studies indicate that *S. uberis* and *B. pumilus* (Wagener *et al*., 2014b, 2015) might represent such bacterial candidates with a hitherto unknown role in uterine pathology. Both bacteria are prevalent in postpartum dairy cows. *S. uberis* is known as an emerging pathogen for mastitis, but has not been described as uterine pathogen. The rarely described *B. pumilus* was found to be associated with endometritis, but it is still unclear if this species is a causing agent for endometritis or is only an opportunistic contaminant in the inflamed endometrium. Results from *in vitro* cell culture studies pointed towards a pro-inflammatory potential of *B. pumilus* on endometrial cells (Gärtner *et al*., 2016).

As shown in some of the above mentioned studies (Santos *et al*., 2011; Santos and Bicalho, 2012; Wagener *et al*., 2015), it has to be underlined that in several cows with clinical signs of metritis and endometritis, neither *E.coli* nor *T. pyogenes* can be detected. In this context, the role of anaerobic bacteria, e.g. *Porphorymonas spp*., as a causing factor as well as a factor for cure rates after antibiotic treatment of metritis needs to be elucidated more in detail (Jeon *et al*., 2017, 2018). Additionally, further research is required to understand the association between uterine diseases and hitherto unknown species (Santos *et al*., 2011; Wagener *et al*., 2014a).

## Molecular mechanism associated with subfertility

Uterine infections as well as cytological endometritis, which are not necessarily congruent, play a crucial role in subfertility in cows. It seems likely that underlying mechanisms for subfertility can be found on molecular level, as reviewed in detail by Sheldon *et al*. (2014).

Several studies found differences in endometrial gene expression of pro-inflammatory mediators, such as cytokines, antimicrobial peptides, acute phase proteins (APP) and prostaglandins between healthy cows and subfertile cows with e.g. subclinical or clinical endometritis (Fischer *et al*., 2010; Drillich *et al*., 2012; Hoelker *et al*., 2012; Peter *et al*., 2015; Ibrahim *et al*., 2016). Upregulated chemokine mRNA expression is required to mediate and direct the PMN influx into the uterine lumen (Zerbe *et al*., 2003). This chemoattractive effect has been found e.g. for Interleukin (IL) 8 and CXCL5 (Fischer *et al*., 2010; Galvão *et al*., 2011). Cytokines IL1A, IL1B, IL6 and TNFα can be regarded as mediators of nonspecific inflammatory processes but are also physiologically upregulated during the early puerperal period (Gabler *et al*., 2010). Highest cytokine and acute phase proteins mRNA expression was observed during the third week postpartum regardless of their health status (Gabler *et al*., 2010; Chapwanya *et al*., 2012). This supports the hypothesis that a certain immune response is essential for postpartum clearance of the uterus. Thus, it would be helpful for research as well as in practice to use pro-inflammatory markers as a diagnostic tool for uterine inflammation.

Other inflammatory mediators associated with uterine diseases are prostaglandins (PG; Arosh *et al*., 2002). In cows with subclinical endometritis 21 to 28 days postpartum concentrations of PGF2α were significantly lower whereas concentrations of PGE2 were higher compared with healthy cow (Baranski *et al*., 2013). It can be hypothesized that this may relate to a switch from PGF2α to PGE2 induced by *E.coli*- derived LPS (Herath *et al*., 2009). Additionally, dysregulated expression of enzymes for PG synthesis (PGES) was found in cows with uterine disorders and reduced fertility (Gabler *et al*., 2009, 2010; Peter *et al*., 2015), e.g. endometrial PGE2 synthase cPGES, which catalyzes the conversion from PGH2 into PGE2, was lower and PGD2 synthase was higher compared with healthy cows. Dys-regulation of PGE synthases might contribute to lower conception rates (Gabler *et al*., 2010). Detailed research is needed to elucidate long term effects of uterine inflammation in the early postpartum period on fertility that manifests later in lactation. In that context, Peter *et al*. (2015) provided some evidence that endometrial inflammation has such a long term effect on PGD2 synthase PTGDS. In this study, endometrial mRNA expression of several pro- inflammatory factors was measured in weekly intervals between 24 and 44 days postpartum. Cows initially diagnosed with subclinical endometritis showed a 3-fold increase in PTGDS expression at the end of the observation period compared with healthy cows. Low levels of PGD_2_ are required for the maintenance of pregnancy (Saito *et al*., 2002). This, however, is only one example of several factors involved in fertility that need further investigation. Furthermore, the difference between the changes in gene expression and the occurrence of clinical signs of disease and future fertility should be elucidated.

In a recent study, we observed differences in gene expression of endometrial epithelial cells between subfertile (repeat breeder) cows and healthy heifers (Wagener *et al*., 2017b). Interestingly, the most pronounced differences were observed for mucins, molecules that are an integral component of the local uterine immune defense. Mucins are anti-adhesive glycoproteins covering epithelial surfaces to protect from bacterial infection and proteolytic attacks. In the uterus, high mucin levels may be desirable after parturition to prevent the establishment of bacterial infections (Sheldon, 2015). In contrary, before embryo implantation, mucin down-regulation of endometrial epithelial cells is required for blastocyst attachment in many species (Braga and Gendler, 1993; Johnson *et al*., 2001). The results of our previous study support the assumption that dys-regulated MUC4 and MUC12 mRNA expression in the uterus may contribute to subfertility in cows (Wagener *et al*., 2017b). Also Kasimanickam *et al*. (2014) suggested a potential relevance of endometrial MUC in subfertile cows.

The effects of an inflamed endometrium on embryo’s quality and survival have been show in several studies (Hill and Gilbert, 2008; Drillich *et al*., 2012; Hoelker *et al*., 2012). From a study with superovulated cows and flushed embryos, we suggested that a certain inflammatory endometrial reaction in terms of PMN influx is beneficial for the number of flushed embryos and, thus, for embryo survival (Drillich *et al*., 2012). Furthermore, changes in endometrial gene expression patterns in cows with and without (subclinical) endometritis included genes involved in cell adhesion and immune modulation (Hoelker *et al*., 2012). There is evidence for an effect of endometrial PMN infiltration on the early stage embryos. In the first week after fertilisation, altered embryonic gene expression profiles between healthy and subclinical endometritic cows were related to membrane stability, the cell cycle and apoptosis (Hoelker *et al*., 2012). Underlying mechanisms for dysregulated gene expression in cows with uterine diseases might be elucidated by expression analyses of microRNAs (miRNAs), regulators of post-transcriptional gene expression. Hailemariam *et al*. (2014) found miRNAs that were differentially expressed in cows with subclinical endometritis, and that are involved in inflammatory responses, cellular proliferation, cell movement, the cell cycle and apoptosis.

## Link between negative energy balance and subfertility

Beside bacterial and viral infections (reviewed by Chastant-Maillard, 2015), several risk factors for endometritis are known, for example calving assistance (Prunner *et al*., 2014), negative energy balance pre- and post-partum (Potter *et al*., 2010), hypocalcaemia, and others (Dubuc *et al*., 2010; Giuliodori *et al*., 2013).

The *in vivo* situation of the reproductive tract environment in ruminants is not only influenced by physiological processes of regeneration, inflammation and infection but is also highly dependent on the metabolic situation of the cow (LeBlanc, 2012).

Because of tremendous needs for energy for milk production and decreasing dry matter intake around calving almost all dairy cows undergo a period of negative energy balance (NEB) in the first 100 days of lactation (LeBlanc, 2010). Cows compensate NEB by lipid mobilization, resulting in increased circulating levels of non-esterified fatty acids (NEFA) and beta- hydroxybutyrate (BHB) and reduced levels of insulin and insulin-like-growth-factor 1 (IGF-1). It is well known that metabolic imbalances during the transition period impair fertility (LeBlanc, 2012). Previous studies have investigated effects of NEB on ovarian function, oocyte and embryo quality (Leroy *et al*., 2008) and uterine health (Chapinal *et al*., 2011). A previous study observed altered gene expression of *IGF* binding protein in the oviduct of cows with NEB; however, the influence of gene expression changes on embryo development were not analyzed (Fenwick *et al*., 2008). It seems that feed restriction during the dry and post- partum period in general influences global gene expression in the oviduct (Valour *et al*., 2013). Thus it is important to consider the metabolic situation of the cow in studies investigating underlying reasons for subfertility. Furthermore, the role of the oviduct in cows with uterine diseases needs further evaluation.

## Summary

In the past decades, our knowledge on uterine infections has increased enormously. Particularly the role and mechanisms of pathogenicity of bacteria have been elucidated, as reviewed by Sheldon *et al*. (2014). Current challenges are the understanding and interpretation of bacterial interactions in the uterine microbiota. Uterine infections cause a cascade of immune reactions in the endometrium which is at least in some ways different from healthy cows that also undergo cellular and molecular changes in the postpartum period. A detailed understanding of these pathogenic mechanisms leading to subfertility might be a key for future prevention and treatment options. Whereas uterine infection and inflammation has been the topic of many studies that elucidated several relations, deeper insights regarding the relationship between uterine diseases and alterations in the oviduct and its functionality is needed to get a broader picture of subfertility in cattle.
